# Treatment of Diabetes by Rennet

**Published:** 1853-03

**Authors:** 


*Treatment of Diabētes by Rennet.*—Dr. Gray, of Glasgow, has lately treated a case of this disease, of at last twelve months' standing, by means of rennet, as prepared for the dairy, in doses of a teaspoonful three times of a day, in which dietetic treatment had been of only partial benefit, and various medicines of little use. He was led to give it from the action of rennet out of the body in converting sugar into lactic acid, which, according to Liebig, is one of the principles of the organic world, which can support respiration by being oxidated in the lungs; while sugar cannot be oxidated by them, and is therefore thrown off as excrementitial by the kidneys.—*N. Y. Med. Times,* Feb. 1853.

